# Oxidative Stress and Cytotoxicity Induced by Co-Formulants of Glyphosate-Based Herbicides in Human Mononuclear White Blood Cells

**DOI:** 10.3390/toxics11120976

**Published:** 2023-12-01

**Authors:** Khadija Ramadhan Makame, Sylvia Nyambeki Masese, Balázs Ádám, Károly Nagy

**Affiliations:** 1 Department of Public Health and Epidemiology, Faculty of Medicine, University of Debrecen, 4032 Debrecen, Hungary; khadija.makame@med.unideb.hu (K.R.M.);; 2 Doctoral School of Health Sciences, University of Debrecen, 4032 Debrecen, Hungary; 3 Institute of Public Health, College of Medicine and Health Sciences, United Arab Emirates University, Al Ain P.O. Box 15551, United Arab Emirates; badam@uaeu.ac.ae

**Keywords:** herbicide, glyphosate, formulation, GBH, co-formulant, adjuvant, cytotoxicity, oxidative stress

## Abstract

The use of genetically modified, glyphosate-resistant crops has led to the widespread application of glyphosate-based herbicides (GBHs), making them one of the most widely used herbicide formulations on the market. To enhance the efficacy of the active ingredient, GBHs used in practice often contain other ingredients marked as inert “adjuvants” or “co-formulants”, the toxic properties of which are poorly understood. The objective of this study was to compare the cytotoxic effects of pure glyphosate, three GBHs (Roundup Mega, Fozat 480 and Glyfos) and two co-formulants commonly used in GBHs as assessed via CCK-8 assay, and the extent of their potential oxidative damage as assessed via superoxide dismutase (SOD) assay, in order to reveal the role of adjuvants in the toxicity of the formulations. Our results showed that glyphosate alone did not significantly affect cell viability. In contrast, GBHs and adjuvants induced a pronounced cytotoxic effect from a concentration of 100 μM. SOD activity of cells treated with GBHs or adjuvants was significantly lower compared to cells treated with glyphosate alone. This suggests that the adjuvants in GBHs are responsible for the cytotoxic effects of the formulations through the induction of oxidative stress.

## 1. Introduction

Glyphosate is one of the dominant active ingredients in today’s plant protection products, used primarily in agriculture and residential areas to eradicate unwanted weeds [1]. Agricultural use of glyphosate-based herbicides (GBHs) has significantly increased since introducing genetically engineered and herbicide-tolerant crops in 1996. Globally, the amount of glyphosate applied by farmers has increased approximately 15-fold, from 51 million kilograms in 1995 to 747 million kilograms in 2014 [2], and it has been estimated that around 385,000 km^2^ of agricultural land is now affected by glyphosate contamination [3]. This herbicide’s widespread and large-scale application has led to its contamination in the environment and edible products [4,5,6]; hence, human exposure is inevitable.

Although glyphosate is the active substance in GBHs, other ingredients, often labeled as “inert”, “co-formulants”, or “adjuvants”, are also added to the formulations to improve the efficacy of the active ingredient. These co-formulants usually vary from one country to another. Unfortunately, the manufacturing industries often consider their identities and exact concentrations as confidential business information, which they are not legally obliged to disclose [7,8]. Consequently, the nature and extent to which these adjuvants contribute to the potentially dangerous effects of GBHs on humans and the environment is often obscured.

The hazards and related risks of GBHs are mainly assessed in terms of the toxicity of the active ingredient, largely neglecting the potential harm of other components and the possible combined toxic effects of mixtures. Most marketed GBHs contain surfactants, including ethoxylated alkylphenols or polyethoxylated tallow amines (POEAs), which increase the solubility of glyphosate by forming micelles and protect it from natural degradation [9]. The presence of co-formulants may promote the penetration of glyphosate into the target plant and the skin of exposed individuals. On the other hand, the supposedly “inert” co-formulants can have biological activity on their own. They may be just as or even more toxic to humans than the active ingredient, or the components might have synergetic effects [7,10]. Previous studies concluded that POEAs or GBHs containing POEAs are more harmful than glyphosate alone and have been reported to significantly increase the acute toxicity of herbicide formulations [8]. Experimental studies indicated that co-formulants, especially POEAs, possess endocrine-disrupting effects and cell membrane damage [10,11,12,13]. Therefore, GBHs containing these surfactants are being removed from the market and replaced by new-generation surfactants that are presumably less toxic than POEAs [14,15].

Over the past few decades, numerous researchers have extensively investigated the harmful effects of glyphosate and GBHs on human cells in vitro, employing various techniques. Glyphosate, when used as an active ingredient, has been found to cause significant damage to DNA in different types of human cells, including HepG2 cells [16], fibrosarcoma cells (HT1080) [17], buccal epithelial carcinoma cells [18] and lymphocytes [19,20]. Recent research involving Burkitt’s lymphoma (Raji) cells exposed to glyphosate suggested that concentrations exceeding 10 mM were lethal to the cells, while physiologically relevant concentrations (≤100 μM) had no cytotoxic effect [21]. Additionally, Kwiatkowska et al. observed increased DNA damage in peripheral blood mononuclear cells (PBMCs) at concentrations ranging from 0.5 to 10 mM [17] and chromosomal damage in lymphocytes occurred at concentrations of ≥6 mM, as evidenced by sister-chromatid exchange [19].

GBHs were found to induce DNA strand breaks in HepG2 cells at concentrations as low as 5 ppm (29.6 μM) [22] and in buccal epithelial carcinoma cells at concentrations of 20 μg/mL (118.3 μM) [23]. In a more recent study by Woźniak et al. [20] the cyto- and genotoxic effects of a GBH (Roundup 360 PLUS) and its active ingredient glyphosate were assessed in PBMCs across a range of concentrations from 1 to 1000 μM. Their findings indicated that Roundup 360 PLUS caused DNA damage starting at 5 μM, substantially reducing cell viability from 50 μM onward. In contrast, glyphosate induced DNA lesions only at concentrations exceeding 500 μM without causing noticeable cytotoxicity [24].

In mammals, including humans, GBHs primarily exhibit cytotoxic and genotoxic effects, triggers inflammation, and disrupts lymphocyte functions and the immune system [25]. A study conducted by Nagy and their colleagues revealed that glyphosate did not cause significant cytotoxicity or genotoxicity within the concentration range of 1 to 1000 μM when used alone. In contrast, commercially available GBHs started causing substantial cell death at concentrations as low as 0.25 mM, along with a significant increase in DNA damage at concentrations of 0.5 mM for Roundup Mega and Glyfos and 0.75 mM for Fozat 480. As a result, the study concluded that the variations in toxicity between GBH formulations and the active ingredient could be explained by the higher cytotoxic activity of the co-formulants present in GBHs or their interactions with the active ingredient glyphosate [26]. Moreover, a recent systematic review of the scientific literature, which focused on comparative studies assessing the toxicity of pesticide active ingredients and their respective formulations, identified twenty-four studies that reported increased toxicity of different pesticide formulations compared to their active ingredients. GBHs were the subjects of ten of these studies; six concluded that Roundup, the most common glyphosate-based formulation, exhibited more significant toxicity than the active ingredient alone [27].

Due to the wide variety of adjuvants and the lack of information on their toxicity and other undesirable characteristics, there is a growing concern that the analysis of glyphosate as an active ingredient alone or with only an insignificant number of GBHs do not provide sufficient information on the possible toxic effects of the formulations used in practice. To adequately protect human health, co-formulants’ toxicity and the interaction between the components of GBHs should be evaluated more systematically. This study aims to investigate whether adjuvants are responsible for the previously observed cytotoxic effects caused by GBHs and whether they exert this effect through the previously hypothesized oxidative damage.

## 2. Materials and Methods

### 2.1. Chemicals and Reagents

Analytic-grade glyphosate (CAS No: 1071-83-6) was purchased from VWR International Kft (Debrecen, Hungary). The three different glyphosate-based herbicides used in this study, which were kindly provided by Hungarian pesticide applicators, are listed below:

Roundup Mega containing 551 g/L or 42% (*w*/*w*) potassium salt of glyphosate (CAS No: 70901-12-1; equivalent to 450 g/L glyphosate) and 7% (*w*/*w*) ethoxylated etheralkylamine (CAS No: 68478-96-6) according to its material and safety data sheet;

Fozat 480 containing 480 g/L or 41% (*w*/*w*) isopropylammonium salt of glyphosate (CAS No: 38641-94-0; equivalent to 360 g/L glyphosate) and <5% (*w*/*w*) hygroscopic substances (CAS No: 66455-29-6) according to its material and safety data sheet;

Glyfos containing 480 g/L or 42% (*w*/*w*) isopropylammonium salt of glyphosate (equivalent to 360 g/L glyphosate) and 9% (*w*/*w*) polyethoxylated tallow amine (CAS No: 61791-26-2) according to its material and safety data sheet.

Alkyl dimethyl betaine (EMPIGEN^®^ BB detergent), a co-formulant of Fozat 480, indicated as Adjuvant A in our study, was purchased from Merck (Darmstadt, Germany). Polyethoxylated tallow amine (ROKAmin SR22), a co-formulant of Glyfos, indicated as Adjuvant B in our study, was kindly provided by PCC Exol SA (Brzeg Dolny, Poland). Cell culture medium and its supplements were obtained from VWR International (Leuven, Belgium). Heparin-containing vacutainers were purchased from B.D. Vacutainer Systems (Plymouth, UK). RIPA Lysis and Extraction Buffer (Cat. number 89900) were obtained from ThermoFisher Scientific (Waltham, MA, USA). Superoxide Dismutase (SOD) Activity Assay kit) (Cat. number ab65354) was purchased from Abcam (Cambridge, UK). Cell Counting Kit-8 (CCK-8, Cat. number ALX-850-039) was obtained from Enzo Life Sciences (Farmingdale, New York, NY, USA).

### 2.2. Cell Culture

Human peripheral whole blood samples were obtained via venipuncture and collected in EDTA-containing vacutainer tubes from three non-smoking, healthy volunteers (males aged 35–37 years) without previous contact with pesticides, mutagens, or carcinogens. Informed consent was obtained from all donors involved in the study. Human mononuclear white blood (HMWB) cells were separated from the erythrocytes via density gradient centrifugation over Histopaque-1077 solution. The buffy coat containing the HMWB cells was aspirated and resuspended in RPMI 1640 medium containing 10% fetal calf serum (FCS), 2 mM L-Glutamine, 100 U/mL penicillin, 100 μg/mL streptomycin, and 250 ng/mL amphotericin. Prior to treatment, the number of cells required for each assay was set up in a Bürker hemocytometer, with each sample containing an equal number of HMWB cells (2 × 10^6^).

### 2.3. Cell Treatment

HMWB cells were treated in the culture medium with increasing concentrations (0.1 μM, 1 μM, 10 μM, 100 μM, 1 mM, 10 mM) of glyphosate alone, the three GBHs (Roundup Mega, Fozat 480, and Glyfos) and the two adjuvants in such a way that the concentrations of glyphosate in GBHs were equivalent, and the concentrations set for the adjuvants were equivalent to their concentrations in the corresponding GBH. The concentrations of GBHs and adjuvants are referred to as glyphosate-equivalent concentrations in this study. Concentrations were chosen based on the results from previous in vitro studies performed on human lymphocytes [20,21]. The stock solutions and dilution series were prepared in RPMI 1640 and adjusted with 1 M NaOH to a pH of 7.2. Various concentrations of glyphosate, GBHs, Adjuvant A and B together with a negative control of RPMI 1640 and a positive control of 100 μM of H_2_O_2_ were added to the cell cultures and incubated at 37 °C for 4 h and 20 h, respectively.

### 2.4. Cytotoxicity Assessment

Cell viability was assessed using the CCK-8 assay. After treatment, cells were centrifuged at 1500 rpm for 2 min. The supernatant was discarded, and 100 μL of CCK-8 reagent (×100 dilution using RPMI 1640 medium) was added to the tubes containing the cells. Cell suspensions were then transferred to a 96-well plate and incubated at 37 °C for 1 h. The absorbance was measured at 460 nm using an Epoch microplate spectrophotometer purchased from BioTek Instruments, Inc. (Winooski, VT, USA). Relative cell viability was quantified by comparing the absorbance (A) of treated cells with that of untreated cells (negative control) with the following formula:

$$Viability\;\left(\%\right)=\frac{A_{sample}}{A_{negative\;control}}\times 100$$

### 2.5. Measurement of Intracellular Oxidative Stress

Superoxide dismutase (SOD) is a vital biological antioxidant enzyme that plays a crucial role in safeguarding cells against oxidative stress. It serves as the primary enzyme responsible for catalyzing chemical reactions aimed at eliminating harmful reactive oxygen species (ROS). One of its essential functions is the dismutation of superoxide radicals into less damaging oxygen and hydrogen peroxide. A decrease in SOD activity is associated with an increase in the production of reactive oxygen species, which can be induced by certain xenobiotics; therefore, SOD activity is a frequently used biochemical index for intracellular oxidative stress assessment after chemical exposures [28,29,30,31].

SOD assay was used to evaluate the oxidative stress level in HMWB cells. The assay utilizes a WST-1 substrate (a tetrazolium salt) to detect superoxide anion radicals generated by xanthine oxidase. WST-1 is transformed into a water-soluble formazan dye upon reduction with superoxide anion. This dye’s production is directly proportional to the activity of xanthine oxidase and is inhibited by the presence of SOD.

The assay was carried out according to the manufacturer’s instructions. In brief, after treatment, cells were centrifuged at 1500 rpm, and the cell pellet was washed with ice-cold PBS. Next, 50 μL of RIPA lysis and extraction buffer supplemented with phosphate and protease inhibitors was added to each tube and left for 30 min in the ice bath. Lysates were ultracentrifuged at 14,000× *g* for 15 min at 4 °C. The supernatants were then transferred to a 96-well plate, and reagents of the SOD assay were immediately added to the samples. The plate was incubated at 37 °C for 20 min.

The reduction of WST-1 to formazan was quantified following the increase in absorbance at 450 nm using an Epoch microplate spectrophotometer (BioTek Instruments, Inc. (Winooski, VT, USA). The percentage of reaction inhibition rate indicates SOD activity and was calculated using the following formula:

$$SOD\;activity\;\left(inhibition\;rate\;\%\right)=\frac{\left(A_{blank1}-A_{blank3}\right)-\left(A_{sample}-A_{blank2}\right)}{\left(A_{blank1}-A_{blank3}\right)}\times 100$$

where A = Absorbance, and blank1, blank2 and blank3 are assay controls.

### 2.6. Statistical Analysis

Data were expressed as mean ± standard error of the means (SEM) of three independently repeated experiments. Cell viability (%) and SOD activity (%) induced by various concentrations of the test agents in repeated experiments were statistically compared to that of untreated cells via one-way ANOVA with Dunnett’s post hoc test using STATA software version 12.0 (College Station, TX, USA). A statistically significant difference was accepted at a 5% significance level.

## 3. Results

### 3.1. Cytotoxicity

The cytotoxicity of the tested substances was evaluated based on the degree of cell survival at each concentration. The viability of cells after treatment with pure glyphosate, GBHs and adjuvants decreased to varying degrees with increasing concentrations and exposure times.

Pure glyphosate exhibited a low level of cytotoxicity (>80% cell viability) after 4 h treatment throughout the tested concentration range. Interestingly, after 20 h of exposure, glyphosate reduced cell viability to 70%.

**Figure 1:**
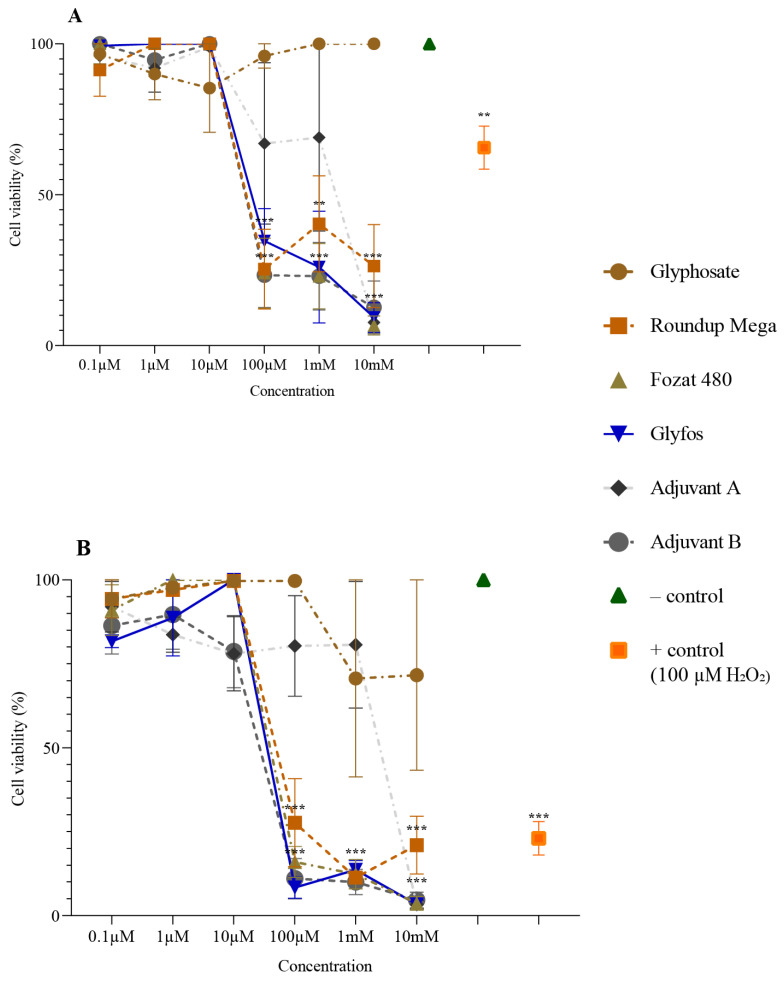
Viability of HMWB cells after 4 h (**A**) and 20 h (**B**) exposure to various concentrations of glyphosate, three GBHs (Roundup Mega, Fozat 480 and Glyfos) and two co-formulants (Adjuvant A and Adjuvant B) detected via CCK-8 assay. The data points indicate the means ± standard error of three repeated experiments’ means (SEM). Statistically significant decrease (** *p* < 0.01, *** *p* < 0.001) in cell viability was determined by comparing the values induced by various doses of glyphosate, GBHs or co-formulants to the background level of untreated cells via one-way ANOVA with Dunnett’s post hoc test.

For GBHs, a statistically significant and marked decrease in cell viability was observed after 4 and 20 h ofexposure to the 100 μM concentration and above. The viability of cells exposed to the co-formulants showed a similar trend to GBHs. The cytotoxic effect induced by Adjuvant B was comparable to that of GBHs. However, 100 μM and 1 mM concentrations of Adjuvant A caused a lower level of cell death than Adjuvant B at both treatment durations (Figure 1).

### 3.2. Superoxide Dismutase Activity

**Figure 2:**
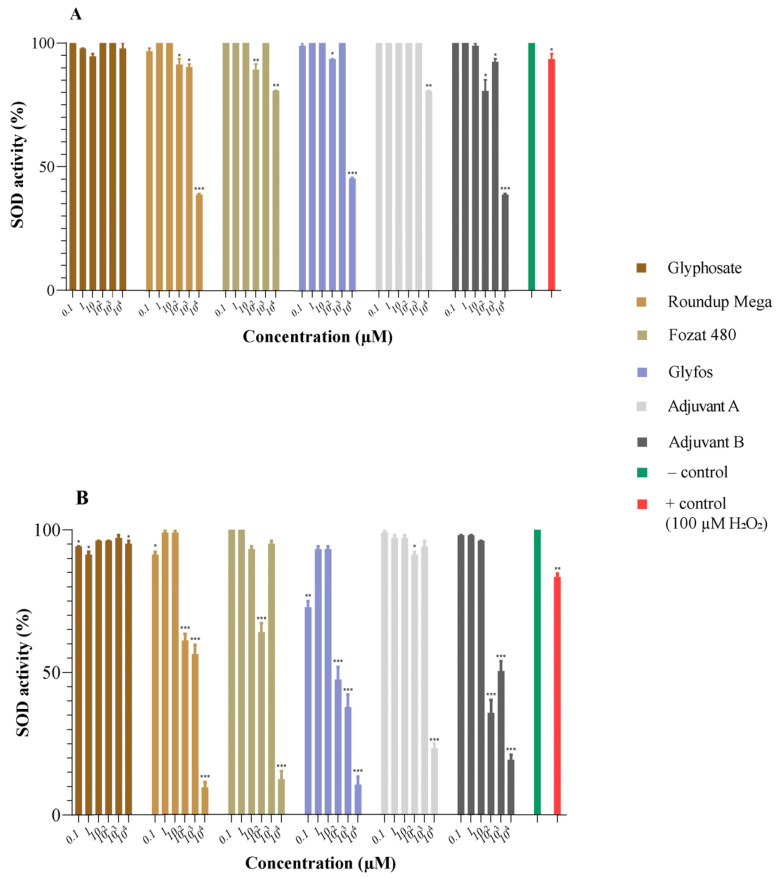
Changes in superoxide dismutase (SOD) activity (%) in HMWB cells induced by 4 h (**A**) and 20 h (**B**) exposure to glyphosate, three GBHs (Roundup Mega, Fozat 480 and Glyfos) and two co-formulants (Adjuvant A and Adjuvant B). Data are means ± standard error of three repeated experiments’ means (SEM). Statistically significant (* *p* < 0.05, ** *p* < 0.01, *** *p* < 0.001) decrease in SOD activity was determined by comparing the values induced by various doses of glyphosate, GBHs and co-formulants to that of untreated cells via one-way ANOVA with Dunnett’s post hoc test.

The action of SOD was decreased in a concentration-dependent manner in the HMWB cells treated with GBHs and co-formulants, especially after 20 h of exposure in the higher concentration range. Glyphosate alone did not substantially affect SOD enzyme activity (>90%). The three GBHs reduced SOD activity to a similar extent, the reduction becoming apparent at concentrations above 100 μM, especially after 20 h of exposure. The pattern of the reduction in SOD activity caused by Adjuvant B was identical to that of GBHs, but the decrease caused by Adjuvant A was found to be moderate compared to Adjuvant B. These results indicate the ability of GBHs and co-formulants to induce oxidative stress (Figure 2).

## 4. Discussion

Various adjuvants are added to pesticide formulations, such as to glyphosate-based herbicides, to increase the effectiveness of the active ingredient [32,33]. Several comparative studies suggested that the toxicity of GBHs is not merely determined by the glyphosate as an active ingredient alone but rather by the co-formulants added to the pesticide formulation and/or their interaction with the active ingredient [8,22]. Due to the extensive use of GBHs, it is necessary to investigate whether these co-formulants influence the toxicity of the formulations used in daily practice. This study investigated whether other ingredients of GBHs play a role in the previously observed cytotoxic effect of the formulations and in the mechanism by which this effect may be mediated.

The overall cytotoxic effect of GBHs increased with increasing concentration. In contrast, the toxicity of glyphosate on its own remained relatively low even at higher concentrations. These findings corroborate previous studies reporting much lower cytotoxic action of glyphosate than GBHs. For instance, the investigation by Gasnier and colleagues reported that the viability of HepG2 human hepatoma cells remained consistently high following acute exposure to glyphosate when compared to GBHs at equivalent concentrations [23]. Similarly, a study comparing the cytotoxicity of pure glyphosate with GBHs showed that the viability of HMWB cells exposed to glyphosate remained consistently above 86% in the 0–1000 μM concentration range [24]. Furthermore, when adult planarians were exposed to glyphosate for a duration of 7 days, lethality was only observed at a relatively high concentration (1000 μM). However, when exposed to GBHs (Roundup^®^ Concentrate Plus and Roundup^®^ Ready-to-Use Weed), lethality was already observed at a much lower concentration (100 μM) [25].

In contrast to glyphosate’s benign effects, all three GBHs reduced cell viability rates below 50% from the concentration of 100 μM. The ability of GBHs to induce higher levels of cytotoxicity than pure glyphosate has been a consistent finding in numerous prior investigations. Nagy and colleagues, for instance, reported that within a four-hour exposure period, GBH formulations resulted in significantly diminished viability of HMWB cells, with rates falling below 23% for Roundup Mega and Glyfos at a concentration of 500 μM. Fozat 480 exhibited similar toxicity but at a concentration of 750 μM [24]. Another GBH, RangerPro, a generic equivalent of Roundup Mega, was found to be ~22 times more cytotoxic than pure glyphosate in the Caco-2 cell line following 24 h exposure [32]. It was also reported that in a study investigating the viability of mouse fibroblast (L929) and human epithelial (Caco2) cells, Roundup Bioflow treatment resulted in lower IC50 than exposure to glyphosate alone [26]. Exposure to Roundup also led to an increased level of cytotoxicity in a human alveolar carcinoma cell line (A549). The proportion of apoptotic cells significantly rose to 72.07% due to 100 μg/mL Roundup treatment in contrast to 2.45% in the control group [34]. Bednářová and colleagues conducted an in vivo study that compared the toxicity of acute and chronic exposure to glyphosate and Roundup. Their observations indicated that the mortality rate of fruit flies (*D. melanogaster*) induced by exposure to 3000 μg/mL Roundup was approximately 52%, compared to 28% after treatment with an equivalent dose of glyphosate. This suggests that Roundup causes significantly higher mortality than glyphosate [35]. 

During the evaluation of the adjuvants’ cytotoxicity, it was evident that Adjuvant A exerted a lower level of cytotoxicity compared to Adjuvant B and to the tested GBH formulations. Adjuvant A, with the IUPAC name alkyl dimethyl betaines, serves as the hygroscopic substance in the Fozat 480 herbicide formulation. It is predominantly employed as a surfactant due to its exceptional foaming properties. As documented in the European Chemicals Agency (ECHA) registries, alkyl dimethyl betaines (in our study, Adjuvant A) do not exhibit any conclusive evidence of genotoxicity, carcinogenicity or reproductive toxicity in both in vivo and in vitro studies. The observed effects in humans primarily include skin and eye irritation [36,37]. Nevertheless, in our study we found evidence of a detectable cytotoxic effect above 100 μM.

Conversely, Adjuvant B, with the IUPAC name polyethoxylated tallow amines, serves as a surfactant in the Glyfos herbicide formulation. Polyethoxylated tallow amines, or POEAs, are a group of chemical compounds employed across various industrial and commercial sectors. Acting as surfactants, they can reduce the surface tension of liquids, enhancing their wetting, spreading and emulsifying characteristics. Tallow amines are typically derived from animal fats which are ethoxylated by the addition of several ethylene oxide units. These non-ionic surfactants are used in herbicide formulations to heighten the effectiveness of the active ingredients [14,35,38]. Multiple studies have highlighted the toxicity associated with polyethoxylated tallow amines. For instance, Mesnage and colleagues tested the toxicity of POEAs, corresponding to Adjuvant B, and found that POEAs exhibited comparatively higher toxicity than glyphosate and Roundup. The LC50 value was approximately 1–2 ppm, making it 100-fold more toxic than Roundup. This assessment was based on the ability of POEAs to inhibit the action of the mitochondrial succinate dehydrogenase enzyme across HEK 293, HepG2 and JEG3 cell lines [39]. In a study by Hao et al., it was concluded that POEA adjuvants in Roundup influenced the apoptotic effect of the formulation. POEAs induced apoptosis in 85.53% of the cells, while the level of apoptosis caused by Roundup was 72.07% (*p* < 0.01) [34]. In yet another study, the outcomes were similar: POEAs had an EC50 of 19.3 mg/L, Roundup Mega exhibited an EC50 of 82.7 mg/L and Roundup Classic had an EC50 of 54.7 mg/L [27]. However, differing results were also reported in other studies where Roundup displayed lower LC50 values (male: 587.7 μg/mL; female: 774.4 μg/mL) in comparison to POEAs (LC50: 1044.9 μg/mL for males and 1322.6 μg/mL for females) when tested with *D. melanogaster* after 72 h of exposure [35]. The disparities in these findings could be attributed to variations in exposure times and the use of higher doses when compared to our study.

The other objective of our study was to elucidate the mechanism of cytotoxicity by assessing the extent of intracellular oxidative stress with superoxide dismutase activity. Our findings demonstrated that glyphosate did not reduce SOD activity to the same extent as GBHs and adjuvants. Specifically, the reduction induced by the GBHs was significantly greater than that of the active ingredient, especially during a 20 h exposure period. The detected SOD activity was also relatively low after treatment with both adjuvants, but slightly higher than in case of the formulations. Notably, Adjuvant A treatment displayed a higher SOD activity at a concentration of 10 mM during a 4 h exposure period, similar to Fozat 480. The decline in SOD activity observed in both GBHs and adjuvants can be attributed to an increased level of superoxide radicals within the samples.

Various studies assessed the mechanism of toxicity of GBHs and co-formulants. Mesnage and colleagues demonstrated that POEA disrupts membrane integrity in human cells even at relatively low concentrations, promoting cellular necrosis and endocrine disruption [39]. Prolonged exposure to pesticides can alter erythrocyte enzyme activity; thus, organophosphate exposure induces oxidative stress and alters the defense mechanism, which is indicated by low levels of SOD activity [40]. A comprehensive review of experimental evidence from research studies suggests that pesticides induce oxidative stress, which is mainly influenced by the induction of reactive oxygen species (ROS) and reactive nitrogen species (RNS) [41]. Oxidative stress has been recognized for its ability to trigger an adaptive response in cells and organisms when encountered at moderate stress levels and low doses. In such conditions, living organisms may activate protective mechanisms to counteract the effects of oxidative stress and maintain cellular and physiological homeostasis. This adaptive response can serve as a defense mechanism against potential harm caused by oxidative stressors. So, moderate stimulations can be beneficial, but high doses can cause inhibitory or toxic effects [11]. The increased cytotoxicity observed in our study, mainly due to exposure to higher concentrations of GBH formulations and adjuvants, may be associated with severe cellular damage caused by oxidative stress due to the induction of reactive oxygen radicals, which may consequently result in the disruption of the cell membrane structure.

Cytotoxicity plays an important role in chemical carcinogenesis, particularly in non-genotoxic mechanisms of carcinogenicity. Non-genotoxic carcinogens do not directly interact with DNA but instead are thought to induce tumor formation by affecting cellular structures and altering the rates of cell proliferation or other processes that heighten the likelihood of genetic errors [42]. Our findings on the role of co-formulants in the cytotoxic effects caused by GBHs may therefore provide valuable input data for the evaluation of the human cancer risk caused by these herbicides.

The general population is exposed to GBHs primarily from food containing residues or the use of household herbicides containing glyphosate, but individuals working in specific occupational environments, such as agriculture or landscaping, where these herbicides are frequently utilized, might face much higher levels of exposure compared to the general population [43]. Despite the widespread use of glyphosate and the rising concerns and debates about its potential adverse health effects in the population, there have been a scarcity of human biomonitoring studies specifically focusing on glyphosate. Research carried out so far has demonstrated that typical exposure levels of GBHs in the general population or in occupational settings do not appear to pose an acute toxicity risk to humans [44]. Although acute toxicity risks are not evident at typical exposure levels, long-term and repeated low-level exposure to GBHs can still lead to potential chronic health effects [45]. Human biomonitoring studies reporting on blood levels of glyphosate are almost absent in the literature. Only two studies have measured glyphosate levels in maternal blood and umbilical cord serum, and in the serum of non-pregnant women. The median value of glyphosate in maternal serum was 17.5 μg/L (0.1 μM). In the umbilical cord, the median glyphosate level was 0.2 μg/L (1.18 nM) [46]. The mean glyphosate level in non-pregnant women was 73.6 ± 28.2 μg/L (0.44 ± 0.17 μM) [47]. Measurements of urinary glyphosate or its main metabolite, aminomethylphosphonic acid (AMPA), also serve as valuable biomarkers for identifying exposure [48]. However, there is currently insufficient toxicokinetic data to establish a direct correlation between the amount of these substances detected in urine and the body’s overall burden of GBHs or the extent of environmental exposure. Despite their utility in indicating exposure, understanding the precise relationship between urinary glyphosate and/or metabolite levels and the actual extent of exposure or the total body burden of GBHs requires further investigation and more comprehensive studies to establish clearer associations.

## 5. Conclusions

This study allowed us to compare the cytotoxicity of glyphosate and GBH formulations with their respective declared co-formulants in human mononuclear white blood cells. The lack of cytotoxicity of the active ingredient glyphosate and the marked cytotoxic effects caused by the co-formulants suggest that the adjuvants may be responsible for the overall toxicity of the GBH formulations and that this effect may be exerted through the induction of oxidative stress. This study confirms previous hypotheses that surfactant adjuvants in GBHs can highly influence the toxicity of these herbicide products.

Our results indicate the importance of a full assessment of the toxicity of adjuvants and formulations as a priority in the pre-market hazard identification and risk assessment of glyphosate-based herbicides, with particular attention paid to the evaluation of their potential late toxic effects (e.g., carcinogenicity).

## Data Availability

The original data presented in the study are included in the article; further inquiries can be directed to the corresponding author.
